# CRISPR/Cas9 gene editing in the West Nile Virus vector, *Culex quinquefasciatus* Say

**DOI:** 10.1371/journal.pone.0224857

**Published:** 2019-11-12

**Authors:** Michelle E. Anderson, Jessica Mavica, Lewis Shackleford, Ilona Flis, Sophia Fochler, Sanjay Basu, Luke Alphey

**Affiliations:** Arthropod Genetics, The Pirbright Institute, Pirbright, Woking, England, United Kingdom; Fundacao Oswaldo Cruz Instituto Rene Rachou, BRAZIL

## Abstract

*Culex quinquefasciatus* Say is an opportunistic blood feeder with a wide geographic distribution which is also a major vector for a range of diseases of both animals and humans. CRISPR/Cas technologies have been applied to a wide variety of organisms for both applied and basic research purposes. CRISPR/Cas methods open new possibilities for genetic research in non-model organisms of public health importance. In this work we have adapted microinjection techniques commonly used in other mosquito species to *Culex quinquefasciatus*, and have shown these to be effective at generating homozygous knock-out mutations of a target gene in one generation. This is the first description of the *kmo* gene and mutant phenotype in this species.

## Introduction

The southern house mosquito, *Culex quinquefasciatus* Say, can be found in abundant numbers in urban and suburban areas from tropical to temperate environments, is geographically widespread and acts as a major vector for a diverse range of different pathogens. *C*. *quinquefasciatus* prefers to feed on birds, but is an opportunistic blood feeder, posing a threat to both human and veterinary health [1, 2] as a major vector for West Nile virus, St. Louis encephalitis virus, eastern equine encephalitis virus, lymphatic filariasis and avian malaria [3].

Due to the lack of specific vaccines or treatments for most of these pathogens, current control strategies focus on reducing the number of mosquitoes, for example through eliminating potential breeding sites or treating them with larvicides [4]. The continuous use of these methods raises concerns of emerging insecticide resistance [5–7] and potential off-target effects of insecticides. It is therefore of vital importance to seek new strategies to control vector species; methods based on genetic manipulation of mosquitoes potentially provide a new class of methods for this purpose [8]. The ability to disrupt or edit genes allows for characterisation and investigation of new gene targets for this purpose, as well as providing a powerful and flexible tool for fundamental research.

One of the successful technologies used for gene editing is the clustered regularly interspaced short palindromic repeats (CRISPR)/Cas9 system, a tool originally derived by exploiting a natural RNA-guided defence system found in bacteria to protect them from invading viral DNA (reviewed in [9]). The CRISPR/Cas9 system is comprised of a Cas9 protein paired with a synthetic single guide RNA (sgRNA). The sgRNA is used to target specific sequence within the genome by complementary base pairing. After binding to the target DNA, the Cas9-sgRNA complex causes a double-stranded break in the DNA. The break is then repaired by either of two pathways: homology-directed repair (HDR) or end-joining, e.g. non-homologous end joining (NHEJ)[10].

CRISPR/Cas9 has been utilised for gene editing in a wide range of insect species since its discovery [11–14]. Attempting gene editing in a non-model species is challenging for many technical aspects including delivery of the Cas9 protein and guide RNA and analysis/characterisation of the resulting mutations, which are typically small insertions or deletions (“indels”). This process can be aided by selection of a target that facilitates recognition of protein disruption without molecular techniques. Previous mosquito gene editing studies have employed targeting of the kynurenine monooxygenase gene (*kmo*), which is essential for eye pigmentation in mosquitoes [15–17]. Direct injections of the Cas9 protein and several sgRNAs into *Aedes aegypti* (L.) embryos successfully generated small insertions and deletions in multiple sites resulting in an easily detectable white eye phenotype in homozygous mutants [16].

In this study CRISPR/Cas9 was successfully utilised to target and generate mutations in the *C*. *quinquefasciatus* genome. Currently we are aware of only two studies based on CRISPR/Cas9 mutagenesis performed in this mosquito [18, 19], and two earlier studies using transposon-mediated transformation [20, 21]; this is the first time the *kmo* gene (CPIJ07147) has been characterised. By acting as a proof of concept, and also providing a potential selectable marker, this research will aid and prompt future CRISPR based gene characterisation studies which will assist both current and future vector control strategies.

## Materials and methods

### Insect rearing

The *C*. *quinquefasciatus* wild type TPRI line (Tropical Pesticides Research Institute, obtained from the London School of Hygiene and Tropical Medicine, London, UK), and gene edited derivative lines, were maintained at 26°C, 60% RH, 12:12 light/dark cycle, and provided 10% sucrose solution *ad libitum*. Artificial blood meals were provided using a Hemotek system (Hemotek Ltd, Blackburn, UK) with defibrinated horse blood (TCS Bioscience, Buckingham, UK), covered with natural sausage casing (salted and dried porcine intestine, Fulks Butchers, Brookwood, UK) and then Parafilm (Bemis, Neenah, WI, USA) membranes. Egg rafts were collected (in a 150ml cup) and hatched into hay infused water. Larvae were fed on guinea pig pellets (Pets at Home Guinea Pig Nuggets, Farnborough, UK).

### CRISPR/Cas9

The *C*. *quinquefasciatus* kynurenine 3-monooxygenase gene was identified in Vectorbase (CPIJ07147), amplified and the sequence confirmed in our lab strain using primers 5’-TCCCCTGACCAAAGAAACCACG -3’ and 5’- CTCGATGTAGTTGTACATGGCCAAATCG -3’. sgRNAs were generated following Basett, *et*. *al*. [11]. Templates incorporating T7 promoters (bold) were amplified using primers: LA935 5’-GAAAT**TAATACGACTCACTATAGG**ACAGTGCGGTCCGCAAGGGTTTTAGAGCTAGAAA-3’, LA936 5’-GAAAT**TAATACGACTCACTATAG****G**CCAGACGTACATCGAGCAGTTTTAGAGCTAGAAA-3’, LA938 5’-GAAAT**TAATACGACTCACTATAG****G**ATCATCATGAACTTGCCGGTTTTAGAGCTAGAAA-3’ and a common reverse primer: 5’-AAAAGCACCGACTCGGTGCCACTTTTTCAAGTTGATAACGGACTAGCCTTATTTTAACTTGCTATTTCTAGCTCTAAAAC-3’ underline indicates sgRNA target. PCR amplicons were purified with the Nucleospin Gel and PCR Clean-up kit (Macherey-Nagel, Düren, DE). sgRNAs were then *in vitro* transcribed with the MEGAscript T7 transcription kit and purified with the MEGAclear transcription reaction clean-up kit (Life Technologies, Waltham, MA, US). Purified sgRNAs were quantified on a Nanodrop (Thermo Fisher Scientific, Waltham, MA, US) to determine concentration and quality assessed on an agarose gel, then aliquoted and stored at -80 until use. Injection mixes for microinjection of embryos consisted of four components: 300 ng/μl nls-SpCas9 protein (PNA Bio, Newbury Park, CA, US) and each of the three sgRNAs at 40 ng/μl in 1X injection buffer [22]. All injection mixes were freshly prepared on ice and centrifuged at 10,000 x *g* and 4°C for at least 10 minutes, to minimize needle blockage.

### Embryo microinjections

A 150ml cup with purified water was introduced to cages on the 5^th^ day post blood meal. Egg rafts were collected for 45–60 minutes, disaggregated, lined up against and orientated parallel to moistened Protran nitrocellulose paper (GE Healthcare, Chicago, IL, US) using a fine bristle paint brush [23]. Lines of embryos were transferred to Scotch Double Sided Tape 665 (3M, St. Paul, MN, US) on a coverslip and covered with Halocarbon oil 27 (Sigma-Aldrich, Dorset, UK). Injections were performed using Sutter quartz needles (1.0mm external diameter 0.70mm internal diameter needles with filaments) drawn out on a Sutter P-2000 laser micropipette puller (Sutter Instrument, CA, US) with the following program: Heat = 729, FIL = 4, VEL = 40, DEL = 128, PUL = 134, LINE = 1. Injections were carried out using a standard microinjection station equipped with a FemtoJet 4x microinjector (Eppendorf, Hamburg, DE). Injected eggs were rinsed with purified water to remove all traces of oil and then recovered onto moist coffee filter paper in a petri dish and maintained at insectary conditions for two days. Hatched first instar larvae and any remaining eggs were transferred to standard rearing conditions two days after injection. Injections detailed in Table 1.

**Table 1:** Embryonic microinjections.

Cas9	sgRNA	Embryos injected	G_0_ adult survivors	G_1_ screened	White-eyed G_1_
300ng/μl PNABio nls-SpCas9 protein	40ng/μl each sgRNA_935, sgRNA_936, and sgRNA_938	1377	157 (11.4%)	1298	35

Embryos were co-injected with three sgRNAs and SpCas9 protein as indicated (see also Methods). Numbers of embryos injected and recovered (G_0_) are shown, together with numbers of white-eyed individuals observed in the next generation (G_1_).

### Screening, imaging, and crossing

**Fig 1:**
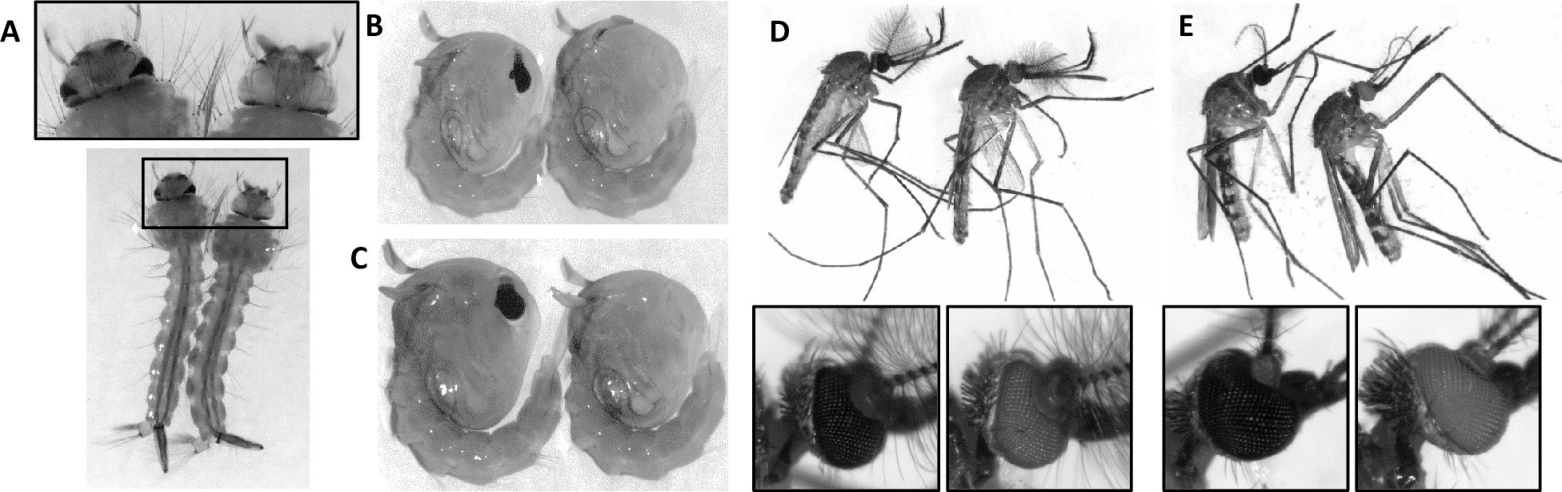
Images of wild-type and *kmo* knock-out mosquitoes. Larvae (A), male pupae (B), female pupae (C), male adults (D), and female adults (E). Images were obtained using a Leica MZ165FC microscope with Leica DFC camera (Leica Biosystems, Wetzlar, Germany).

All adult G_0_ survivors were pooled together and blood fed. G_1_ late stage larvae and pupae were screened for white eyes. The 23 white eyed G_1_ females were then pool crossed to 46 wild-type males, and the 12 white eyed G_1_ males were pool crossed to 36 wild-type females in separate 15x15x15 cm cages, and blood fed. After three ovipositions the white eyed G_1_ progeny were snap-frozen in liquid nitrogen for molecular analysis. The heterozygous and thus black eyed G_2_ individuals were sibling crossed and white eyed G_3_ progeny were used to establish the colony. Images in Fig 1 were obtained using a Leica MZ165FC microscope with Leica DFC camera (Leica Biosystems, Wetzlar, Germany).

### Predictions and confirmation of indels

Genomic DNA was extracted from individual G_1_ mosquitoes using the NucleoSpin Tissue kit (Macherey-Nagel, Düren, Germany). PCR was performed using Q5 High-Fidelity DNA Polymerase or Phusion Hot Start Flex 2X Master Mix (New England Biolabs, Ipswich, MA, US) with primers:

5’-TGTAGAAGTTGGTGAACGGCA-3’ and 5’-TGTTGAGTGAGTTTTCGCGATG-3’. PCR amplicons were verified by agarose gel electrophoresis, purified using the NucleoSpin Gel and PCR Clean-up kit (Macherey-Nagel, Düren, Germany) and Sanger sequenced (Eurofins, Ebersberg, Germany). Chromatograms were analysed using the ICE web tool available from Synthego (Redwood City, CA, US) https://ice.synthego.com/#/. The recently published inDelphi web tool [24] was used to predict likely mutations resulting from editing with the two sgRNAs which gave mutations. The top 10 predicted indels are presented in Tables 2 and 3. Complete predictions can be found: https://indelphi.giffordlab.mit.edu/single_mESC_pl~Ff9H5g4WqrtlfBaPMzIL4Lbkf~e6eyh1pHnVgznZJcMxMiwF_-_77 for sgRNA935 and https://indelphi.giffordlab.mit.edu/single_mESC_rGpragvGMG7BJ4fe9a59YuGaqtquLSsfT3hkFm5_GTG_60 for sgRNA936.

**Table 2:** Summary of inDelphi predictions at target site with gRNA 935: GTACAGTGCGGTCCGCAAGG.

Alignment	Category	%
CGACGGAGCGTACAGTGCGGTCCGCA|AGGAGGTCATCAAACGTCCGGGGTAC	Reference	-
CGACGGAGCGTACAGTGCGGTC----|--------ATCAAACGTCCGGGGTAC	12-bp deletion	20.8%
CGACGGAGCGTACAGTGCGGTCCGCA|-GGAGGTCATCAAACGTCCGGGGTAC	1-bp deletion	11.4%
CGACGGAGCGTACAGTGCGGTCCGCA**A**AGGAGGTCATCAAACGTCCGGGGTAC	1-bp insertion	11.4%
CGACGGAGCGTACAGTGCGG------|---AGGTCATCAAACGTCCGGGGTAC	9-bp deletion	6%
CGACGGAGCGTACAGTGCGGTCCGCA|---------TCAAACGTCCGGGGTAC	9-bp deletion	4.4%
CGACGGAGCGTACAGTGCGGTCCG--|--------------------GGGTAC	22-bp deletion	4.3%
CGACGGAGCGTACAGTGCGGTCCG--|--GAGGTCATCAAACGTCCGGGGTAC	4-bp deletion	2.8%
CGACGGAGCGTACAGTGCGGTCCGCA|----GGTCATCAAACGTCCGGGGTAC	4-bp deletion	2.6%
CGACGGAGCGTACAGTGCGGTC----|AGGAGGTCATCAAACGTCCGGGGTAC	4-bp deletion	2.3%
CGACGGAGCGTACAGTGCGGTCCG--|---AGGTCATCAAACGTCCGGGGTAC	5-bp deletion	2.2%

**Table 3:** Summary of inDelphi predictions at target site with gRNA 936: CAGACGTACATCGAGCACGG.

Alignment	Category	%
TACGACTTCAGCCAGACGTACATCGA|GCACGGCTACCTGGAGCTGTGCATTC	Reference	-
TACGACTTCAGCCAGACGTACATCG-|-----GCTACCTGGAGCTGTGCATTC	6-bp deletion	14.4%
TACGACTTCAGCCAGACGTACATCGA**A**GCACGGCTACCTGGAGCTGTGCATTC	1-bp insertion	10.9%
TACGACTTCAGCCAGACGTACA----|---CGGCTACCTGGAGCTGTGCATTC	7-bp deletion	8.2%
TACGACTTCAGCCAGACG--------|-----GCTACCTGGAGCTGTGCATTC	13-bp deletion	6.5%
TACGACTTCAGCCAGACGTACATCG-|-CACGGCTACCTGGAGCTGTGCATTC	2-bp deletion	5.2%
TACGACTTCAGCCAGACGTACATCGA|--ACGGCTACCTGGAGCTGTGCATTC	2-bp deletion	5.1%
TACGACTTCAGCCAGACGTAC-----|----GGCTACCTGGAGCTGTGCATTC	9-bp deletion	5.0%
TACGACTTCAGCCAGACGTACATCGA|---CGGCTACCTGGAGCTGTGCATTC	3-bp deletion	3.8%
TACGACTTCAGCCAGACGTACATCG-|GCACGGCTACCTGGAGCTGTGCATTC	1-bp deletion	3.3%
TACGACTTCAGCCAGACGTACATCGA|-CACGGCTACCTGGAGCTGTGCATTC	1-bp deletion	3.3%

## Results and discussion

### Selection of target site

**Fig 2:**
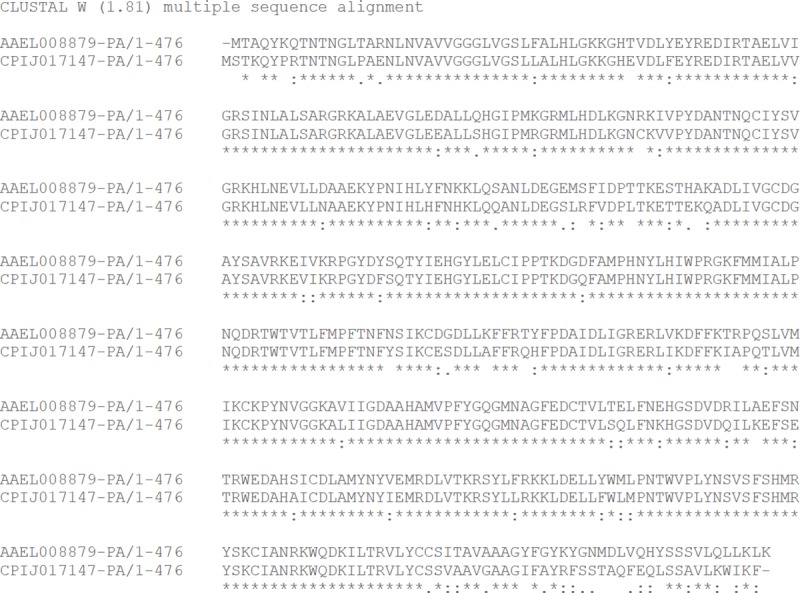
Clustal W alignment of Kmo proteins of *A*. *aegypti* and *C*. *quinquefasciatus*. Orthologue alignment generated within Vectorbase for *A*. *aegypti* AAEL008879 1-to-1 orthologue in *C*. *quinquefasciatus* genome.

To identify a target likely to result in a white-eyed phenotype, the region homologous to exon 5 of the *A*. *aegypti kmo* gene, previously demonstrated by Aryan *et*. *al*. [16] to disrupt protein function when targeted was selected. After confirming the sequence of this region in our lab strain the *A*. *aegypti* and *C*. *quinquefasciatus* exon 5 amino acids were aligned (Fig 2) to ensure the targets were analogous. CHOPCHOP v2 [25] was used to identify sgRNAs within exon 5 of *C*. *quinquefasciatus kmo* with no perfect matches to another location in the genome.

### *C*. *quinquefasciatus* microinjections

In preparation for embryo injections, the adult cages (all adults at least 5 days post eclosion) were blood fed either overnight or for two consecutive days up to one week prior to the intended injection day. Cages to be used for injection were reared in an incubator with an opposite day/night cycle so that injections in the day occurred during the night time cycle. Oviposition cups with purified water were introduced to cages and females were allowed access for up to one hour at which point the cup was removed. Pale grey/green egg rafts were transferred from the surface of the water to a droplet of water in a petri dish, then gently disaggregated with a fine bristle paint brush. A moistened narrow strip of nitrocellulose membrane was used to provide an edge against which the eggs were aligned in parallel [23]. Approximately 30–40 eggs at a time were aligned, then the excess moisture was blotted away with paper towelling and the eggs were transferred to a piece of double stick tape on a plastic coverslip. The eggs were allowed to desiccate slightly then covered with Halocarbon oil to prevent further desiccation. In a preliminary experiment to optimise embryo survival, injection directly into the posterior through the periplasmic space (our standard *A*. *aegypti* injection method) resulted in a lower survival rate (5/167, 3%) than when injected laterally (30/179, 17%).

Embryo injections were performed over the course of two days, using several cages of adults in order to collect a sufficient number of synchronised embryos. After injection, embryos were rinsed thoroughly with water to remove residual traces of halocarbon oil. A fine paintbrush was used to remove the embryos from the tape, which were transferred onto a moistened coffee filter within a plastic petri dish and maintained at insectary conditions. The following day, water was added to the dish submerging the filter paper. On the second day post injection, L1 larvae and remaining eggs were transferred to rearing trays. A sample of the G_0_ survivors were examined for eye pigmentation, mosaicism was observed in approximately 40% of the sample, indicating successful injection of functional CRISPR/SpCas9 complexes.

To increase the likelihood of identifying double knock outs and thus white eyed G_1_ individuals, G_0_ males were mated to G_0_ females. G_1_ progeny were screened as late larvae or pupae when eye colour is most readily identified (Fig 1). Out of over 1300 G_1_ progeny screened, 2.7% (n = 35) were identified as having a complete loss of eye pigmentation (Table 1). White eyed G_1_ adults were then crossed to WT. After collecting 3 ovipositions of G_2_ progeny the G_1_ individuals were collected for sequence analysis. The G_2_ progeny were then crossed to their siblings and white eyed G_3_ individuals were used to establish the colony, which has now been maintained for 15+ generations.

### Analysis of indel mutations

**Fig 3:**
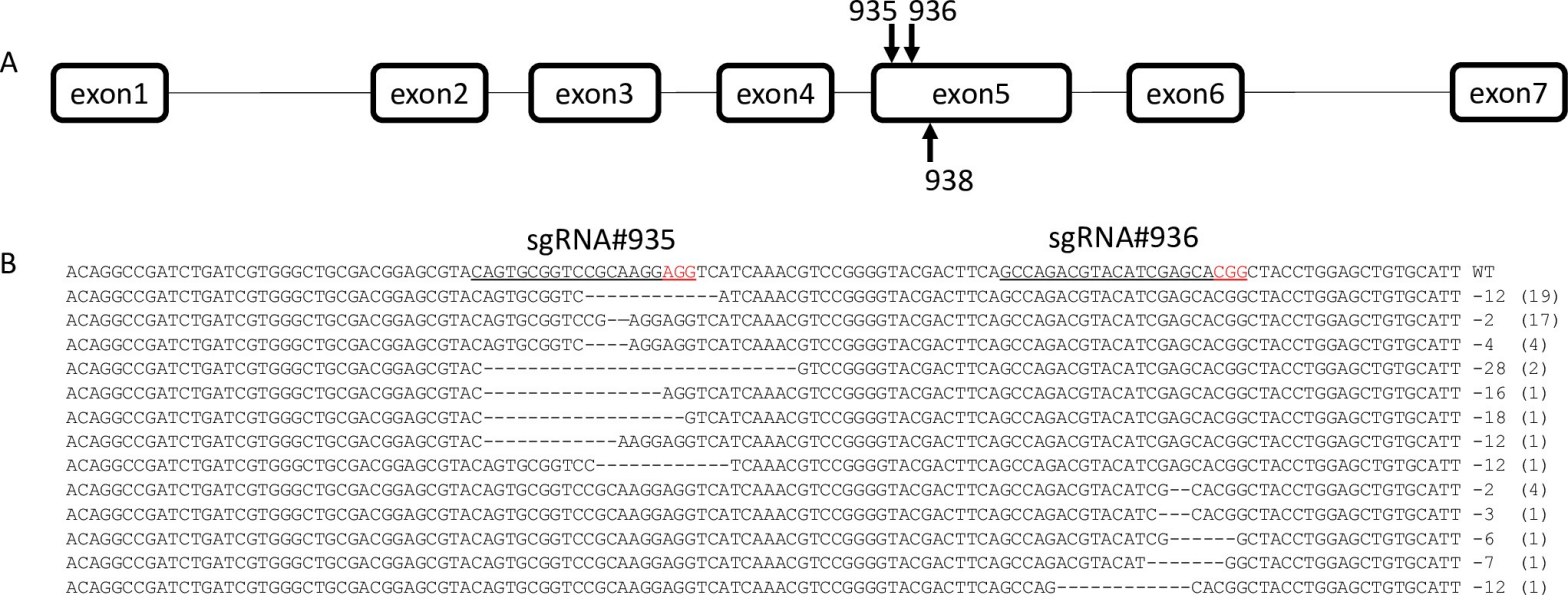
*Culex quinquefasciatus kmo* gene and mutations observed. Structure of CPIJ017147 as annotated in Vectorbase, sequences of exon 2 through 5 were confirmed in our lab strain and three sgRNA targets in exon 5 were selected (A). White eyed G_1_ individuals were collected for genomic DNA extraction, PCR, and sequencing of the *kmo* locus. All sequenced mutations are presented (B) followed by the size of the indel, and in brackets the number of sequenced alleles with that mutation. All sequenced alleles contained deletions derived from sgRNAs 935 and 936.

Of the three sgRNAs injected there was a strong bias in mutations around the predicted site for one of the three. Of the 54 sequenced alleles, 8 appeared to result from sgRNA 936. All other mutant alleles align closely to the cut site for sgRNA 935. There was no evidence of any mutations resulting from sgRNA 938. Although these three sgRNAs target a relatively small region (within 167bp) observed cut rates were highly variable. This further supports the use of multiple target sites/sgRNAs when a knockout phenotype is desired. Interestingly, the white eyed phenotype was observed from both in and out of frame mutations (Fig 3).

The inDelphi web tool [24] was used to predict mutations and their frequencies resulting from the two sgRNAs which generated indels in this study. This machine learning algorithm was trained on a large dataset from several mammalian cell lines and we were interested in its accuracy when applied to mosquitoes. Using mouse Embryonic Stem Cells (mESC) as a starting point, inDelphi was able to predict the mutations we observed for sgRNA 935. The top 10 predicted indels for this sgRNA are presented in Table 2. The highest predicted mutation is a 12bp deletion, predicted at 20.8% frequency. This was indeed the most frequent mutation observed for this sgRNA, found in 35% of sequenced alleles. However, the next two predicted indels, a 1bp deletion and a 1bp insertion were not observed in our samples. The other two most frequent indels observed for this sgRNA, a 2bp deletion (31% of sequenced alleles) and a 4bp deletion (7.4%) were predicted by the algorithm, but at much lower frequencies than were observed (predicted 1.5% and 2.3% respectively).

The highest frequency predictions for sgRNA 936 (Table 3) were a 6bp deletion and a 1bp insertion at 14% and 11%. While we did observe one allele with the 6bp deletion (1.9%), we did not observe any of the other top 10 predicted indels. The most frequent indel observed was a 2bp deletion at 7.4%, which was predicted at 5.2% frequency. The remaining observed indels were not predicted by the software at all. 1bp insertions comprised a major class of products observed in human cells used for training the inDelphi algorithm [24], however we did not observe any insertions, in our samples, perhaps indicating a difference in the repair pathways between human and mosquito cells.

Another potential class of mutations, larger deletions based on simultaneous cuts at two or more sgRNA target sites, was not observed. This is somewhat surprising if all cuts are independent. However, it has been suggested that close proximity of multiple target sites limits Cas9’s ability to cleave adjacent sites due to Cas9-dependent supercoiling [26, 27].

Despite the recent record of gene editing in various mosquito species there are still few reports of genetic manipulation (transgenesis or gene editing) in *Culex* spp. Allen *et al*. successfully obtained transgenics with transposon-mediated transformation using a *Hermes-*based vector[21]. In contrast, several attempts to generate transgenics with *piggyBac*-based vectors using multiple donor constructs and different sources of *piggyBac* transposase, injected as plasmid [28] or *in vitro* transcribed mRNA [29], we did not recover any transgenics. In these experiments, the minimum transformation efficiency was estimated as 0.005, compared to 0.059 for *A*. *aegypti* reported in Gregory et. al., [30]. This important vector species has been neglected in the research community, however CRISPR/Cas9 gene-editing along with optimised rearing and microinjection procedures will allow for important progress in studying this species. Recently bioinformatics tools, like inDelphi, have been described but the utility of machine learning based algorithms beyond the scope of their datasets remains to be seen. The usefulness of such tools across species and from cell line to *in vivo* also has yet to be determined. This study is the first use of the inDelphi tool in mosquitoes and while the most abundant indel for one sgRNA was predicted, the indels generated by the other sgRNA were not. Expanding the data sets used to train these algorithms may ultimately increase their utility but currently their predictive capacity seems only modestly generalizable beyond the cell line or species used.

The overall frequency of white-eyed mosquitoes observed in the G_1_ was 2.7% (35/1300). *kmo* mutations are recessive, so appearance of white eyes implies both alleles are non-functional. Assuming these were disrupted independently, i.e. in the germlines of the male and female G_1_ parents, this would imply a single-allele mutation rate of 0.16 (0.027^0.5^). This may be an underestimate as sequence changes that did not result in loss of gene function would not have been detected by this method, though our identification of a 12bp in frame deletion resulting in a white-eyed phenotype indicates that at least some in frame deletions in the target region could be detected. It is possible that some white-eyed offspring may instead have resulted from zygotic cleavage of one or more alleles in the G_1_ embryos, due to inheritance of SpCas9-sgRNA complexes from an injected G_0_ parent, however we did not observe the mosaic eye phenotype one would expect if this occurred at a significant rate. Single-allele gene editing rates in the 10% range would allow identification of mutant individuals by PCR or other molecular methods following very modest numbers of embryo injections. For genes without an obvious phenotype, homology-directed repair (HDR) could be investigated allowing use of a dominant marker for screening. Our findings suggest that mutational analysis of genes of interest in this species may now be practical.
